# RamA upregulates the ATP-binding cassette transporter *mlaFEDCB* to mediate resistance to tetracycline-class antibiotics and the stability of membranes in *Klebsiella pneumoniae*

**DOI:** 10.1128/spectrum.01728-24

**Published:** 2024-12-31

**Authors:** Xiaoyu Zhao, Yixin Zhang, Mohan Ju, Yang Yang, Haoqi Liu, Xiaohua Qin, Qingqing Xu, Min Hao

**Affiliations:** 1 Institute of Antibiotics, Huashan Hospital, Fudan University 159397 , Shanghai, China; 2 Key Laboratory of Clinical Pharmacology of Antibiotics, National Heath Commission, Shanghai, China; 3 Institute of Microbes and Infections, Huashan Hospital, Fudan University 159397 , Shanghai, China; The University of North Carolina at Chapel Hill, Chapel Hill, North Carolina, USA

**Keywords:** *Klebsiella pneumoniae*, antimicrobial resistance, membrane stability, RamA, *mlaFEDCB*

## Abstract

RamA is an intrinsic regulator in *Klebsiella pneumoniae*, belonging to the AraC family of transcription factors and conferring a multidrug resistance phenotype, especially for tetracycline-class antibiotics. The ATP-binding cassette transporters MlaFEDCB in bacteria play essential roles in functions essential for cell survival and intrinsic resistance to many antibiotics. We found deletion of *ramA* resulted in a fivefold decrease in the transcriptional levels of the *mlaFEDCB* operon. After complementation with ramA, the transcriptional levels were comparable to those of wild-type strain. Furthermore, an electrophoretic mobility shift assay showed that RamA could bind to the promoter region of *mlaEFDCB* operon, which confirmed RamA is an activator of *mlaEFDCB* operon. The *mlaEFDCB* operon could mildly mediate resistance to the tetracycline family of antibiotics under RamA regulation. The MIC (minimum inhibitory concentration) of tigecycline decreased fourfold, and the MIC of doxycycline, minocycline, and eravacycline decreased twofold after *mlaE*-knockout. The *ramA*- and *mlaE*-knockout strains exhibited greater sensitivity to sodium dodecyl sulfate (SDS)-EDTA than the wild-type. Growth of Δ*ramA* cells was severely compromised in 0.25/0.5% SDS and 0.55 mM EDTA, and this sensitivity was restored by complementation with *ramA* and *mlaE*. This study demonstrates that RamA can directly regulate the *malEFEDCB* operon, thereby mediating resistance to tetracycline-class antibiotics, contributing to the stability of bacterial membranes in *K. pneumoniae*. We identified a novel signal pathway in which RamA mediates multidrug resistance of *K. pneumoniae*, leading to new ideas for the development of novel antimicrobial therapeutics, therefore deserving further comprehensive study.

**IMPORTANCE:**

Multidrug-resistant and extensively drug-resistant *Klebsiella pneumoniae* have emerged as significant global health concerns resulting in high mortality rates. Although previous research has investigated the maintenance of lipid asymmetry (Mla) pathway, the extent to which it mediates antimicrobial resistance in *K. pneumoniae* and the underlying upstream regulatory mechanisms remain unclear. In this study, we sought to determine at the molecular level how the AraC-type global regulator RamA directly regulates mlaFEDCB, which mediates resistance to tetracycline-class antibiotics and the stability of bacterial membranes in *K. pneumoniae*.

## INTRODUCTION

*Klebsiella pneumoniae* is one of the most prevalent bacterial pathogens responsible for a diverse spectrum of hospital-acquired infections (1). Multidrug-resistant and extensively drug-resistant *K. pneumoniae* have emerged as significant global health concerns resulting in high mortality rates and substantial hospital expenses (2). Currently, the most common mechanism of tetracycline resistance in *K. pneumoniae* involves efflux pumps (AcrAB/OqxAB) (3), which in gram-negative bacteria are responsible for multidrug resistance, including the resistance nodulation division family, major facilitator superfamily, and the ATP-binding cassette (ABC) superfamily (4).

The global transcriptional activator RamA, classified within the AraC/XylS family of regulatory proteins, plays a vital role in the resistance and pathogenic mechanisms of *Enterobacteriaceae* with wide-ranging consequences for several classes of transcription genes (5, 6). In our previous study, we found ramA could increase the transcription level of both efflux pumps *acrAB* and *oqxAB* which lead to the resistance of tigecycline in *K. pneumoniae* (3). Further transcriptome sequencing has been used to analyze the transcriptomes of wild-type *K. pneumoniae* and *ramA*-knockout strains. Despite acrAB and oqxAB, the results revealing a notable reduction in the transcription levels of the *mlaFEDCB* gene cluster (*mlaF*, *mlaE*, *mlaD*, *mlaC*, and *mlaB*) in a *ramA*-knockout strain compared with those in the wild-type strain.

Maintenance of lipid asymmetry (Mla) pathway belongs to the ABC transporters which comprise an extensive family of membrane transport proteins that perform a range of functions essential for cell survival in harsh environments and provide intrinsic resistance to multiple antibiotics (7). A previous study has reported that the MacAB-TolC ABC-type tripartite multidrug efflux pump contributes to macrolide antibiotic resistance and virulence in *Escherichia coli* (8). To date, however, there have been no reports on the mechanisms of resistance mediated by ABC transporters in *K. pneumoniae*. This ABC transport system has been described as the Mla pathway in *K. pneumoniae* and the VacJ/Yrb ABC transport system in other gram-negative bacteria (9, 10). Gram-negative bacteria Mla proteins (Mla A-F) that play important roles in phospholipid transport (11). This system comprises three main components: (i) an inner membrane ABC transporter complex, MlaFEDB; (ii) an OM complex, MlaA-OmpC/F; and (iii) a soluble periplasmic protein, MlaC, which has been proposed to shuttle phospholipids between MlaFEDB and MlaA-OmpC/F. The MlaFEDB complex comprises four different proteins, including MlaD, a membrane-anchored protein from the mammalian cell entry protein family that forms a homohexameric ring in the periplasm (12, 13), and MlaE (also known as YrbE), a predicted integral inner membrane ABC permease that occupies a key position within the complex. In the absence of MlaE, assembly of the phospholipid transporter is impaired, rendering it biologically non-functional. The complex also includes MlaF, an ABC ATPase, and MlaB, a sulfate transporter and anti-sigma factor antagonist domain protein that is potentially involved in regulatory processes (13).

Recent studies have highlighted the key role of the Mla pathway in conferring polymyxin resistance to *Pseudomonas aeruginosa* (14), comparatively few studies have been conducted on resistance mechanisms mediated by the Mla pathway. In *Haemophilus parasuis*, mutations in the Mla pathway have been observed to result in resistance to multiple antimicrobial classes (15). Furthermore, YrbB has been established to play a protective role against the lethal effects of quinolones in *E. coli* (16). However, the extent to which the Mla pathway mediates antimicrobial resistance in *K. pneumoniae* and the underlying upstream regulatory mechanisms remain unclear.

On the basis of these findings, we investigated the role of RamA in regulating the expression of *malFEDCB* leading to etracycline-class antibiotics and membrane homeostasis in *K. pneumoniae*.

## RESULTS

### RamA regulates expression of the *mla*FEDCB operon

**Fig 1:**
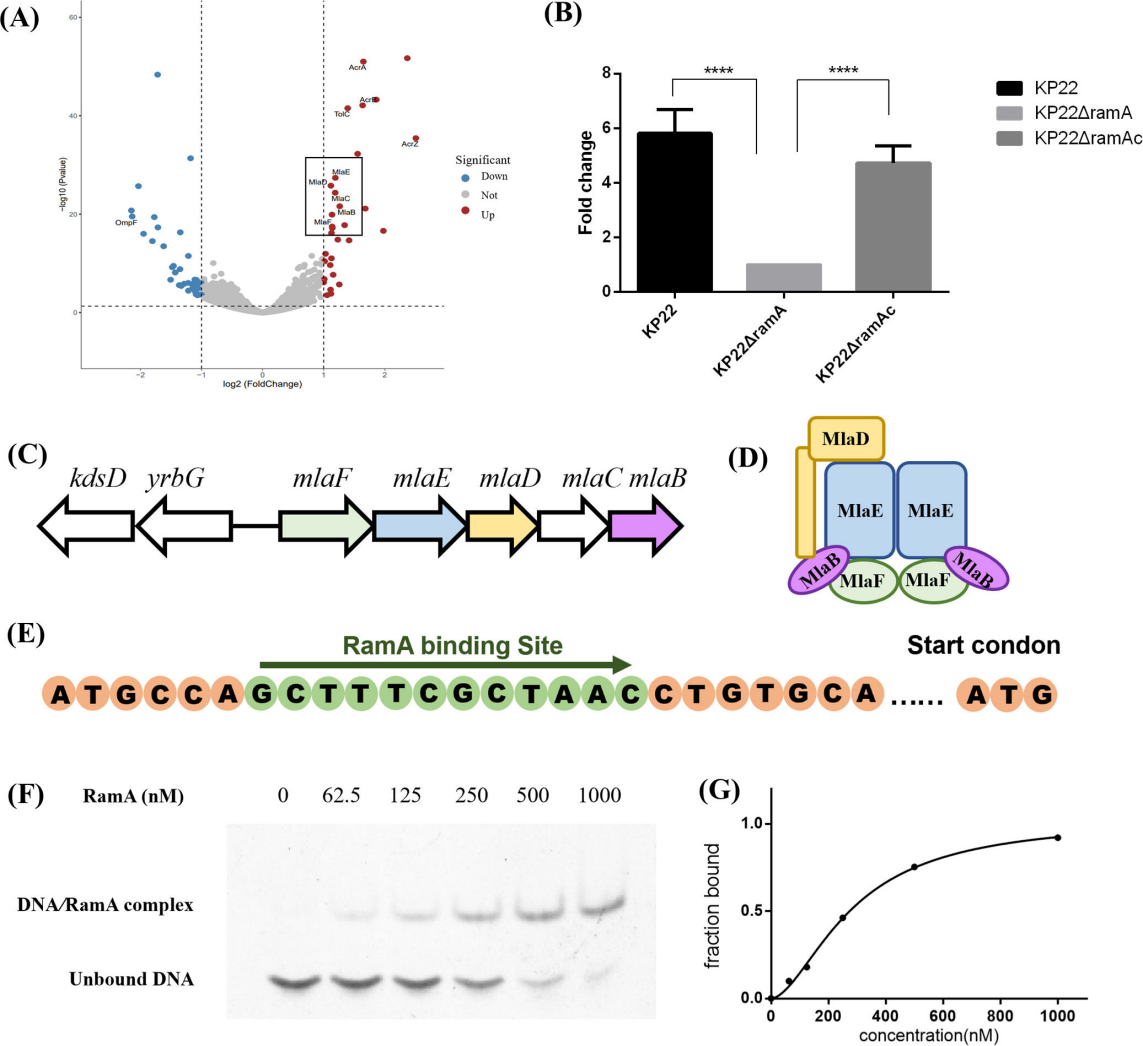
RamA regulates expression of the mla*FEDCB* transposon. (**A**) A volcano plot of upregulated and downregulated genes. (**B**) Transcriptional expression levels of *mlaE* in wild-type *K. pneumoniae*, *ramA*-knockout strain (KPΔ*ramA*), and *ramA* complementation strain (KPΔ*ramAc*). Compared with the KP22Δ*ramA* isolate, the transcription level of *mlaE* was fivefold higher in the wild-type KP22 isolate. Following *ramA* complementation, the levels of *mlaE* transcription were similar to those of wild-type KP22. ****P < 0.0001. (**C**) Schematic representation of the mla*FEDCB* transposon. Each gene is represented by an arrow indicating the direction of transcription with the gene names above. (**D**) Overall structural model of the MlaFEDB transporter. (**E**) Binding of RamA at the DNA promoter region. (**F**) EMSA using purified RamA protein. RamA reduces the migration of *mlaE* promoter DNA. Lanes 1 and 3, pmol promoter DNA of *mlaE*; lanes 2–6, different concentrations of RamA. (**G**) Affinity curve between RamA and promoter DNA.

KP22 is a tigecycline-resistant clinical isolate that, compared with *K. pneumoniae* ATCC 13883, overexpresses RamA. In this study, we initially knocked out *ramA* in the KP22 isolate, and transcriptome sequencing was performed to analyze the transcriptomes of wild-type *K. pneumoniae* and *ramA*-knockout strains, with the R package “limma” being was used to identify genes that were differentially expressed in the 5,081 profiles. Compared with the wild-type strain, the transcription level of *mlaF*, *mlaE*, *mlaD*, *mlaC*, and *mlaB* genes decreased approximately fourfold in the *ramA*-knockout strain based on analysis using volcano plots (Fig. 1A). We further verified changes in the *mlaE* gene by performing quantitative reverse transcription (qRT)-PCR, which revealed the transcription levels of *mlaE* to be fivefold higher in the wild-type KP22 isolate than those in the KP22Δ*ramA* isolate. As consequence of *ramA* complementation, *mlaE* transcription recovered to levels comparable to those in the wild-type strain KP22 (Fig. 1B). The genes *mlaF*, *mlaE*, *mlaD*, *mlaC*, and *mlaB* are contiguous and co-transcribed in the genome, collectively forming the mla*FEDCB* gene cluster (Fig. 1C). The overall structural model of the MlaFEDB transporter is shown in Fig. 1D.

### RamA binds to the promoter region of *mlaFEDCB* regulon

Given that AraC family transcription factors bind to marbox sequences, we analyzed promoter sequences from approximately 59 bp upstream of the *mlaFEDCB* start codon. Further analysis of the promoter sequences indicated the presence of a specific binding site for RamA (5ʹ-CCAGCTTTCGCTAAC-3ʹ) within the *mlaFEDCB* promoter region (Fig. 1E). To establish whether RamA acts directly on *mlaFEDCB*, we performed electrophoretic mobility shift assays (EMSAs), the results of which indicated that RamA bound to the promoter region of the *mlaFEDCB* operon (Fig. 1F and G), on the basis of which, we conclude that RamA is an activator of *mlaFEDCB*.

### MlaE influences the level of antibiotic resistance in clinical *K. pneumoniae* strains under RamA regulation

Our team has previously reported that RamA regulates both the AcrAB and OqxAB efflux pumps, thereby mediating tigecycline resistance in *K. pneumoniae*. In this study, we found that in response to RamA regulation, the *MlaFEDCB* operon mediates moderate resistance to tetracycline family antibiotics. Moreover, following the knockout of *mlaE*, we detected a fourfold reduction in the MIC (minimum inhibitory concentration) of tigecycline and twofold reductions in the MICs of doxycycline, minocycline, and eravacycline (Table 1).

**TABLE 1:** Antimicrobial susceptibility proﬁle of *Klebsiella pneumoniae* knockout and complemented strains

Antimicrobial agent	MIC^*a*^				
	KP-WT	KPΔ*ramA*	KPΔ*ramA*c	KPΔ*mlaE*	KPΔ*mlaE*c
Doxycycline	8	0.5	8	4	8
Minocycline	16	1	16	8	16
Tigecycline	8	≤0.5	8	2	8
Eravacycline	2	0.125	2	1	2
Piperacillin/tazobactam	16	≤4	8	16	16
Ceftazidime	0.5	≤0.12	1	0.5	0.5
Levofloxacin	1 S	≤0.12	1	1	1

^*a*^ Minimum inhibitory concentrations were determined using the broth microdilution methods and are expressed as μg/mL.

### Loss-of-function RamA and MlaE results in an increase in sodium dodecyl sulfate (SDS)-EDTA sensitivity

**Fig 2:**
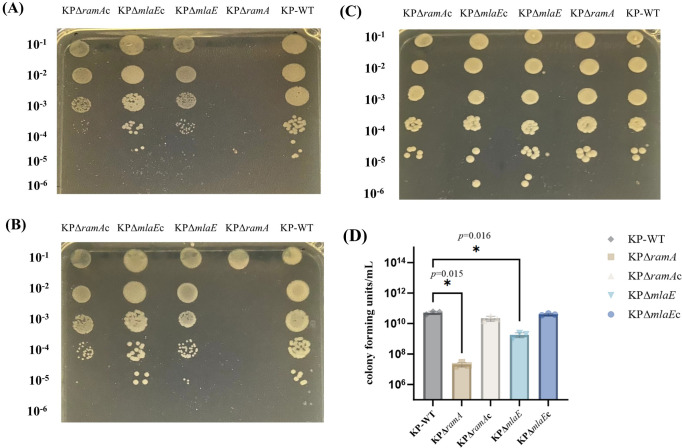
A map of *Klebsiella pneumoniae* ramA and mlaE genes and SDS-EDTA sensitivity assay. (**A**) LB agar medium supplemented with 0.5% SDS and 0.55 mM EDTA. (**B**) LB agar medium supplemented with 0.25% SDS and 0.55 mM EDTA. (**C**) LB agar medium. Approximate dilutions are shown on the left. (**D**) Colony-forming units per milliliter on agar media containing 0.5% SDS and 0.55 mM EDTA.

We subsequently sought to determine whether the deletion of *ramA* and *mlaE* would have similar effects on *K. pneumoniae*. To assess the sensitivity of the KP22 knockout and wild-type strains to detergents, bacterial cultures were diluted and spotted onto Luria-Bertani (LB) agar plates containing 0.25% SDS, 0.5% SDS, and 0.55 mM EDTA. Each dilution was also spotted onto LB agar plates without SDS or EDTA to control for the number of bacteria added per spot (Fig. 2C). The *ramA-* and *mlaE*-knockout strains were accordingly found to exhibit greater sensitivity to SDS-EDTA than the wild-type strain (Fig. 2A and B). In particular, the growth of Δ*ramA* cells was severely compromised by 0.25/0.5% SDS and 0.55 mM EDTA, whereas the sensitivity to SDS-EDTA was restored following complementation with the *ramA* and *mlaE* genes.

Colony-forming units (CFU) on agar media containing 0.5% SDS and 0.55 mM EDTA were counted, and the CFU per milliliter (CFU/mL) were calculated. The *ramA-* and *mlaE*-knockout strains showed significantly lower colony-forming units than wild-type, while complementation strains restored this phenotype.

## DISCUSSION

In this study, we sought to determine at the molecular level how the AraC-type global regulator RamA directly regulate*s mlaFEDCB*, which mediates resistance to tetracycline-class antibiotics, and the stability of bacterial membranes in *K. pneumoniae*.

The AraC family of transcription factors are widely present among bacteria in the *Enterobacteriaceae*, with the most common members being RamA, MarA, SoxS, and Rob. Given the similarity in their DNA binding sequences, these transcription factors regulate a wide range of common downstream target genes (17). RamA is an important resistance gene in gram-negative bacteria, and research to date on the mechanisms of antimicrobial drug resistance mediated by RamA has tended to focus on two main pathways. In the first of these, expression of multiple efflux pumps, such as AcrAB and OqxAB, is upregulated to expel drugs (18), whereas in the second, expression of the sRNA *micF* is upregulated to reduce the expression of the OM porin OmpF, thereby preventing the entry of drugs into cells (19–21). Previous studies have reported that the MarA protein, which is a member the same transcription factor family as RamA, can regulate lipid trafﬁcking and OM integrity by binding to the *mlaFEDCB* operon, thereby influencing resistance to tetracycline antibiotics such as doxycycline and minocycline in *E. coli* (22). The findings of the present study provide initial evidence that RamA mediates resistance to tetracycline family antibiotics by regulating the expression of *mlaFEDCB* in *K. pneumoniae*. Few relevant studies have been reported regarding the activity of the Mla pathway in mediating bacterial resistance (14–16). Munguia et al. found that the Mla pathway regulates OM dynamics in the human pathogen PA (14). Disruption of the Mla pathway gene *vacJ* was found to sensitize PA to the host cathelicidin antimicrobial peptide LL-37. Loss of *vacJ* expression reduced PA survival and virulence in a murine model of lung infection caused by *H. parasuis*. An Mla pathway mutation revealed four- to eightfold reductions in the MIC values forcefotaxime, cefaclor, levofloxacin, and tilmicosin, and only a slight reduction in those for enrofloxacin and florfenicol. Furthermore, a study has revealed that YrbB plays a protective role against the lethal action of quinolones in *E. coli* mediated via a hydroxyl radical-independent and toxin-antitoxin-dependent mechanism, thereby identifying it as a potential target for antimicrobial enhancement.

Mla pathway in gram-negative bacteria plays an important role in phospholipid transport (11). The pathway comprises six proteins, including the ABC transporter MlaFEDB, periplasmic protein MlaC, and OM protein MlaA, associated with osmoporin, which functions in monitoring the direction of phospholipid transport (23, 24). The findings of recent studies have highlighted the essential role of the Mla pathway in conferring resistance to OM permeabilization and facilitating host innate immune clearance in PA (14). Additionally, the Mla pathway plays a pivotal role in regulating different aspects of bacterial physiology, including flagellin expression, motility, formation of OM vesicles, adhesion to epithelial cells, and induction of intestinal inflammatory responses (14, 25, 26). Furthermore, studies have indicated that deletion of Mla pathway genes enhances OM permeability and sensitivity to OM stressors such as SDS and EDTA in *Stenotrophomonas maltophilia*, *P. aeruginosa*, and *E. coli* (11, 18, 27). Our findings in the present study revealed that the RamA-mediated Mla pathway plays an important role in maintaining membrane stability in *K. pneumoniae*, which is consistent with the findings of previous studies. One limitation of this study is that although we performed *malE* gene knockout, we did not assess the effects of deleting other genes in the Mla pathway. We can only assume that by knocking down *mlaE*, MlaFEDB is unable to form a complex and therefore has no biological function.

In conclusion, our findings in this study emphasize that RamA plays a key role in regulating the integral inner membrane ABC permease *mlaE*, thereby mediating resistance to tetracycline-class antibiotics and contributing to the stability of bacterial membranes in *K. pneumoniae*. We have accordingly identified a novel signaling pathway in which RamA mediates multidrug resistance in *K. pneumoniae*, which will provide a basis for the development of novel antimicrobial therapeutics that warrant further comprehensive study.

## MATERIALS AND METHODS

### Bacterial strains and plasmids

*K. pneumoniae* KP22, a clinical isolate collected at Huashan Hospital in Shanghai, was isolated from the urine of a patient with an uncomplicated urinary tract infection. Identification of the isolate was confirmed using a VITEK 2 Compact System (bioMérieux, Lyon, France).

### RNA sequencing analysis

Total RNA was extracted from bacterial cells as previously described (28), and subsequent RNA sequencing was performed commercially by Shanghai Biotechnology Corporation (Shanghai, China). To determine genes that were differently expressed between wild-type *K. pneumoniae* and the *ramA*-knockout strain (KPΔ*ramA*), we used the R package “limma.” Genes with a log2-fold change and adjusted values <0.05 were identified as being differentially expressed. The false discovery rate was controlled using the Benjamini-Hochberg method, and the R package “ggplot2” was used to construct volcano plots (29).

### Transcriptome sequencing and qRT-PCR

To determine the transcriptional expression levels of *ramA* and *mlaFEDCB* in KP22, KPΔ*ramA* and KP*cramA* strain, we performed qRT-PCR. *rrsE* was used as an endogenous reference gene. Total RNA was extracted and qRT-PCR was performed (28) using SYBR Premix *Ex Taq* (TaKaRa, Dalian, China) in an ABI ViiA 7 real-time PCR system (Thermo Fisher Scientific, USA). The reactions were repeated in triplicate, and fold changes in the expression of these genes were calculated as previously described (30). Furthermore, *K. pneumoniae* ATCC 13883 was used as the reference standard for gene expression analysis. The experiment was repeated for three times. Welch’s *t*-test as implemented by GraphPad PRISM 6 was used for data analysis and *P*-values smaller than 0.05 were considered statistically significant.

### Gene knockout and complementation

The chromosomal gene *mlaE was* knocked out using a strategy derived from that used by Datsenko and Wanner (31). Briefly, the plasmid pKOBEG, which mediates λRed homologous recombination induced by l-arabinose (0.5% wt/vol), was electroporated into KP22. To construct the *mlaE-*deletion mutant, primers for upstream F/R (PCR product 500 bp) and downstream F/R (PCR product 500 bp) regions of *mlaE* were designed. The upstream and downstream fragments were inserted into the plasmid pMD-18T, which confers hygromycin resistance and has the same restriction enzyme sites as the PCR product. The fragment was then used to transform KP22 cells via electroporation, and the resultant transformants were screened using PCR. Complementation of wild-type *mlaE* was conducted in KP22Δ*mlaE* (deletion of *mlaE*), using pHSG398 vector derivatives. Empty pHSG398 plasmids were used as a negative control.

### EMSA

Wild-type *ramA* linked to an N-terminal polyhistidine tag was expressed in *E. coli* BL21(DE3), and the RamA transcription factor protein was purified using the denaturing and refolding method, as previously described (32). Subsequently, EMSAs were performed to determine the interaction between the *mlaE* promoter and RamA. Binding reactions (20 mL) containing 0.25 µM cy3-labeled promoter and different concentrations of RamA (ranging from 0 to 1 µM) were mixed on ice and incubated for 30 min. The final reaction buffer contained 20 mM Tris, 12% glycerol, 125 mM NaCl, and 5 mM MgCl_2_. Subsequently, 10 µL of each reaction mixture was loaded on polymerized 5% Tris-glycine native polyacrylamide gels, and electrophoresed at 100 V for 60 min in a cold room. The results were analyzed by visualizing and quantifying the fluorescent signals of Cy3 using ImageJ and GraphPad PRISM 6.

### Antimicrobial susceptibility testing

The MICs of several antibiotics were determined for wild-type *K. pneumoniae. ramA*-knockout (KPΔ*ramA*), *ramA* complementation (KPΔ*ramAc*), *mlaE*-knockout (KPΔ*mlaE*), and *mlaE* complementation (KPΔ*mlaEc*) strains. Antimicrobial susceptibility testing was conducted using the broth dilution method, and the results were interpreted based on the Clinical and Laboratory Standards Institute (CLSI) document M100-S31. The MICs of piperacillin-tazobactam, ceftazidime, levofloxacin, doxycycline, and minocycline were determined according to CLSI standards. Moreover, the MICs of tigecycline for *K. pneumoniae* was determined according to the Food and Drug Administration criteria (susceptible, ≤2 mg/L; intermediate, 4 mg/L; resistant, ≥8 mg/L), whereas eravacycline with an MIC of ≤2 was categorized as susceptible.

### SDS-EDTA sensitivity assays

SDS-EDTA sensitivity assays were performed as described by Malinverni and Silhavy (18). Brieﬂy, *K. pneumoniae* cultures were grown to the mid-log phase [optical density 0.5 to 0.8 at 650 nm (OD_650_)]. Subsequently 2 µL of serial dilutions were spotted onto LB agar containing 0.25% or 0.5% SDS and 0.55 mM EDTA, and the plates were incubated overnight at 37°C. The overnight culture was collected and spread onto LB agar medium. Colonies were counted between 12 and 15 h of incubation. The experiment was repeated for three times. Welch’s *t*-test as implemented by GraphPad PRISM 6 was used for data analysis, and *P*-values smaller than 0.05 were considered statistically significant.
